# An Extended Duration of the Pre-Operative Hospitalization is Associated with an Increased Risk of Healthcare-Associated Infections after Cardiac Surgery

**DOI:** 10.1038/s41598-020-65019-8

**Published:** 2020-05-14

**Authors:** Patrick Sulzgruber, Sebastian Schnaubelt, Lorenz Koller, Günther Laufer, Arnold Pilz, Niema Kazem, Max-Paul Winter, Barbara Steinlechner, Martin Andreas, Tatjana Fleck, Klaus Distelmaier, Georg Goliasch, Aurel Toma, Christian Hengstenberg, Alexander Niessner

**Affiliations:** 1 Division of Cardiology, Department of Internal Medicine II, Medical University of Vienna, Vienna, Austria; 2 Department of Emergency Medicine, Medical University of Vienna, Vienna, Austria; 3 Division of Cardiac Surgery, Department of Surgery, Medical University of Vienna, Vienna, Austria; 4 Department of Anesthesia, General Intensive Care and Pain Management, Medical University of Vienna, Vienna, Austria

**Keywords:** Epidemiology, Epidemiology

## Abstract

Nosocomial infections are a common complication in clinical practice with major impact on surgical success and patient outcome. The probability of nosocomial infections is rapidly increasing during hospitalization. Therefore, we investigated the impact of a prolonged pre-operative hospital stay on the development of post-operative infection. Within this prospective observational study, 200 patients scheduled for elective cardiac surgery were enrolled. Patients were followed during hospital admission and screened for the development of nosocomial infection. Logistic regression analysis was used to assess the impact of a prolonged pre-operative hospital stay on the development of infection. A total of 195 patients were suitable for the final analysis. We found a strong and direct association of the duration of pre-operative hospital stay and the number of patients developing infection (+23.5%; p = 0.006). Additionally, the length of patients’ pre-operative hospital stay was independently associated with the development of post-operative nosocomial infection, with an adjusted OR per day of 1.38 (95%CI: 1.02–1.86; p = 0.036). A prolonged pre-operative hospital stay was significantly associated with the development of nosocomial infection after cardiac surgery. Those findings need to be considered in future clinical patient management in order to prevent unnecessary antibiotic use and potential harm to patients.

## Introduction

Healthcare-associated infections (HAIs) are a common complication in clinical practice associated with increased hospitalization-related costs and worse patient outcome. Recent data of the World Health Organization (WHO) revealed a global burden of HAIs of up to 15% among all hospitalized individuals, with more than 30% in intensive care patients^1^. Of alarming importance, the development of HAI proved to be directly associated with both short and long-term mortality in affected individuals^2^. It is well established that the probability of HAI development rapidly increases during hospital stay in general, and data also suggests the pre-operative length of stay to be a risk factor^3,4^. Especially extensive surgical procedures such as cardiac surgical interventions proved to have an increased risk for the development of infectious disease during post-operative patient care^5^, in particular for mediastinitis^6^. Despite the achievements of disinfection, sterilization and antibiotic therapy, the incidence of HAIs is still increasing within the western society. This subsequently underlines a major need to identify potential influencing and easily adjustable factors for HAI development. Whereas it is well-known that infection influences the length of hospital stay, vice-versa data on the impact of the duration of the pre-operative hospitalization has not been fully investigated so far^7^. It seems intuitive that an extended pre-operative hospitalization increases the individual exposition to potential pathogens, posing an elevated risk for HAIs in the post-operative state^8^. Therefore, we aimed to investigate the impact of a prolonged pre-operative hospital stay on HAI development after cardiac surgery.

## Methods

In this prospective cohort study, a total of 200 patients with structural heart disease and/or stable coronary vessel disease (CVD) scheduled for elective cardiac valve and/or coronary artery bypass graft surgery (CABG) at the Department of Cardiac Surgery of the Medical University of Vienna (Austria) were enrolled. Enrollment was conducted between 5/2013 and 4/2015 at the time of the patients’ hospital admission. All patients were continuously followed up during the entire hospitalization and screened for the development of any nosocomial infection. HAI was defined in accordance to established criteria of the International Nosocomial Infection Control Consortium (INICC) as an evident infection with the associated clinical symptoms developed after day three of hospitalization^9^. The study protocol complies with the Declaration of Helsinki and was approved by the local ethics committee of the Medical University of Vienna (EK 1110/2013). All patients gave written informed consent for participation. Data reporting was performed according to the STROBE and MOOSE guidelines.

### Data acquisition

Patient data were assessed at the time of study inclusion and inserted into a pre-defined record abstraction form. Values of routine laboratory parameters were assessed at the time of hospital admission, immediately after surgery and prior to hospital discharge. Levels of C-reactive protein (CRP) were assessed daily during the hospital stay to elucidate the baseline level and the maximum increase after surgery in accordance to the local laboratory standards of the Medical University of Vienna (Roche Diagnostics, Switzerland).

### Statistical analysis

For statistical analysis patients were stratified into groups depending on their length of pre-operative hospital stay in ≤ 2 days (surgery on time), 2–7 days (delayed surgery) and >7 days (extensive delay of surgery). Categorical data are presented as counts and percentages and were analyzed using a test for linear association (Maentel–Haenszel chi-square test). Continuous data are illustrated as medians and the respective interquartile range using Kruskal–Wallis test for testing within the subgroups. A logistic regression model for the association of the duration of the patients’ pre-operative hospitalization on the development of HAI was performed. Continuous variables were log-transformed prior to their analysis in the regression model in order to adjust data more closely to normal distribution. Data were presented as odds ratio (OR) and the respective 95% confidence interval (CI) per one day increase for continuous variables as the standard outcome value for logistic regression models. The multivariate model was adjusted for “type of surgery” and “Euro Score II”. Two-sided p-values of <0.05 were chosen as statistically significant. Statistical analysis was performed using SPSS 22.0 (IBM, USA).

## Results

Five out of 200 enrolled participants (2.5%) already presented with elevated CRP values and/or fever prior to the cardiac surgical procedure and were therefore excluded for the final analysis, resulting in 195 individuals. After stratification according to the duration of the pre-operative hospital stay, we found that 28.7% of patients (n = 56) received surgical intervention on time (<2 days). One third of all individuals (32.3%; n = 63) had a prolonged pre-operative stay (3–7 days) and 39.0% had an extensive delay (>7 days) – with a total median duration of pre-operative hospitalization of 14 days (Supplementary Table). Patients were followed-up daily during the pre-operative course of hospitalization. There were no events detected that might explain both an increased risk for HAI or prolongation of the pre-operative hospitalization.

**Figure 1:**
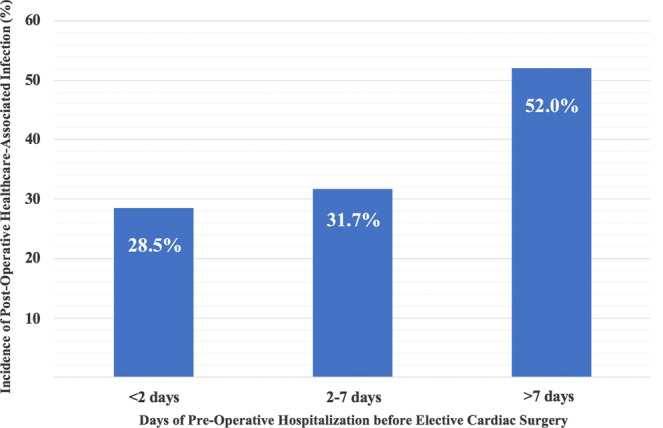
The incidence of post-operative healthcare-associated infections significantly increased with increasing prolongation of a pre-operative hospitalization (p = 0.006).

A total of 75 patients (38.5%) developed any infection after cardiac surgery. We found a strongly increased overall infection rate with prolongation of pre-operative hospitalization when comparing patients that received a surgical intervention on time and individuals with an extensive delay (28.5% vs. 52.0%; p = 0.006; Fig. 1). Interestingly, stratified by the respective types of infection, there was a significant absolute increase in surgical site infection (0.0% vs. 7.9%; p = 0.034) and pneumonia (7.1% vs. 14.5%; p = 0.049). No difference was found for central venous catheter infections (p = 0.245) and urinary tract infections (p = 0.346). Moreover, the duration of the pre-operative hospitalization proved to be directly associated with HAIs with a crude OR per day of 1.45 (95%CI: 1.08–1.95; p = 0.013). The observed effect remained stable even after adjustment for potential cofounders, showing a strong and independent association with HAIs (adjusted OR per day of 1.33 [95%CI:1.01–1.12; p = 0.039]).

Out of the entire study population, a total of 6 individuals died after the surgical procedure. We found that the duration of the pre-operative hospitalization was significantly associated with in-hospital mortality with a crude OR of 1.13 per day (95%CI: 1.04–1.22; p = 0.003). However, this effect was lost after adjustment in the multivariate model (Table 1).

**Table 1:** Nosocomial Infections and In-Hospital Mortality for the entire study population and stratified by days of pre-operative hospitalization and Logistic Regression Analysis for the Association of the Total Pre-Operative Hospital Stay on Infection and Mortality.

	Total	≤2 days	3–7 days	>7 days	p-value	OR (95% CI)	p-value	*Adj. OR (95%CI)	p-value
Any Infection, n (%)	75 (38.5)	16 (28.5)	20 (31.7)	39 (52.0)	**0.006**	1.45 (1.08–1.95)	**0.013**	1.33 (1.01–1.12)	**0.039**
Surgical Site Infection, n (%)	9 (4.6)	0 (–)	3 (4.8)	6 (7.9)	**0.034**	1.57 (0.76–3.22)	0.222	ns.	
Central Venous Catheter Infection, n (%)	37 (19.0)	9 (16.1)	10 (15.9)	18 (23.7)	0.245	1.19 (0.84–1.71)	0.319	ns.	
Pneumonia, n (%)	17 (8.7)	4 (7.1)	2 (3.2)	11 (14.5)	**0.049**	1.27 (0.76–2.09)	0.358	ns.	
Urinary Tract Infection, n (%)	24 (12.3)	6 (10.7)	6 (9.5)	12 (15.8)	0.346	1.19 (0.78–1.83)	0.417	ns.	
Mortality, n (%)	6 (2.6)	0 (—)	0 (—)	6 (6.6)	0.019	1.13 (1.04–1.22)	**0.003**	1.19 (0.98–1.17)	0.110

Categorical data are presented as counts and percentages, continuous data as medians and IQRs. Categorical data are analyzed using a test for linear association (Maentel–Haenszel chi-square test), continuous data using Kruskal–Wallis test for testing within the subgroups. Logistic regression model for the association of the total duration of the patients’ pre-operative hospital stay on the development of infections and major adverse events within the total study population. Odds ratios (OR) for continuous variables refer to an increase per day.

*The multivariate model was adjusted for: Type of surgery and EuroScore II.

## Discussion

Through our analysis, we could show a strong and independent association of the duration of pre-operative hospitalization and the development of post-operative HAIs in the special population of patients undergoing elective cardiac surgery. It is well known that HAIs have an impact on the need for prolonged hospitalization, higher readmission rates and increased mortality from both a short- and also long-term perspective^10,11^. Moreover, HAIs pose a considerable economic impact on healthcare systems, and their prevention is cost-effective^12^. Leung *et al*. could demonstrate that patients with a longer preoperative length of stay are more prone to mediastinitis after cardiac surgery^6^. However, the association of the length of pre-operative hospital stay as potential factor for general infection control in cardiac surgery patients has not yet been fully described.

Evidence-based approaches were implemented in daily clinical routine, aiming at a reduction of infection rates and improvement of the primary patient outcome^5,13–15^. Many of those interventions follow an antimicrobial approach. In accordance to local referral patterns, nasal decontamination and perioperative antibiotic prophylaxis with an increased dose are routinely performed^16,17^. Similarly, the rate of post-operative sternal infections could be significantly reduced with local antibiotic administration^18^.

Patients developing HAI pose a general need for an aggravated antibiotic therapy. Facing an increasing dissemination of multi-resistant bacteria among healthcare institutions – mainly due to unjustified and potential unnecessary antibiotic treatment – the prevention of HAIs poses an essential benefit against the development of antibiotic resistances. Considering the global burden of HAIs and its strong association with patient outcome, identification of modifiable risk factors for infection development are crucial both from a patient-centered-, and a global environmental perspective of nosocomial infection control.

In this regard, a wide range of predisposing factors for the development of HAIs have been identified in literature, including diabetes, impaired renal function, obesity or female gender^19–21^. Those factors are non-modifiable patient characteristics that increase the odds for HAI development. Therefore, the identification of adjustable influencing variables seems of utmost importance to ensure patients’ safety and favorable outcome - such as the limitation of the duration of the pre-operative hospitalization of patients.

Given the fact that all enrolled individuals in this analysis had medical approval for the respective elective surgery at the time of admission, a delay based on a poor medical condition cannot be assumed within this analysis. The observation that patients presenting with a higher EuroScore II experienced a prolonged pre-operative hospitalization, relates probably to the increased pre-operative work-up (consisting of transthoracic echocardiography, aortic CT-scan, lung function testing, and coronary angiography). However, an optimization of variables such as smoking status or glycemic control that is already achieved prior to hospital admission could help reducing hospital stay duration^22^.

In terms of pre-operative risk stratification, we found that patients spending longer time periods in the hospital in the pre-operative phase showed a tendency towards a higher Euro Score. Because of the potential association with patient outcome, we adjusted our regression model. Here, this result still proved to be significant. The Euro Score as a surrogate for “riskier” surgery is a potentially adjustable risk factor - for instance this could be addressed through a flexible team formation with only experienced surgeons operating on patients with high score values. Therefore, the composition of the surgical team could possibly influence patients’ duration of surgery, hospital stay times, and also infection rates.

At the same time, a lack of both operating room and intensive care capacities for a large patient number increases the preoperative waiting time. This could yet again lead to an increased infection rate due to various reasons such as prolonged fasting and following poor nutritional status^6^. Considering the results of this analysis, a pre-hospitalization of more than 7 days is not justifiable given the increased risk of infection. Organizational requirements and referral patterns considering a pre-operative work-up of especially high-risk patients should be adapted to these results and implemented in heart-team meetings of cardiologists and cardiac surgeons for ideal patient care.

### Limitations

The major limitation of this analysis represents its single center setting that alters the overall picture of our results. However, taking into consideration that patients were enrolled during a two-year observation period, we might overcome a potential selection bias (e.g. only depicting the work of a specific team that is more prone to postoperative infection). Moreover, our patient collective only underwent cardiac surgery – therefore, our results cannot be applied to a general population of surgical patients. Another potentially limiting factor is that we could not provide details on the exact timing or causative agent of infection. This could have given additional information and data to discuss^23^. Lastly, other factors apart from the listed could have influenced an increased risk for HAI, for example deconditioning in the elderly, were not taken into account and could have biased results. Such other potentially modifiable factors should be subject of future research.

## Conclusion

The duration of the pre-operative hospitalization is a potentially adjustable factor in clinical practice. Therefore, an optimization of internal resources as well as the enhancement of operating room and intensive care capacities may significantly reduce the rates of nosocomial infections, extensive antibiotic drug use and hospitalization-related mortality in patients undergoing cardiac surgery. This can increase favorable long-term patient outcome.

## Supplementary information

Supplementary information
