# Supplementary material for: An Extended Duration of the Pre-Operative Hospitalization is Associated with an Increased Risk of Healthcare-Associated Infections after Cardiac Surgery
Source: Sci Rep. 2020 May 14;10:8006. doi: 10.1038/s41598-020-65019-8 (PMC7224271; doi:10.1038/s41598-020-65019-8)
Supplement: Supplementary file 1 — Supplementary information [file 41598_2020_65019_MOESM1_ESM.docx]

**An Extended** **Duration of the Pre-Operative Hospitalization is Associated with an Increased Risk of Healthcare-Associated Infections after Cardiac Surgery**

Patrick Sulzgruber, MD, PhD, MBA *(PS)*^1^*****; Sebastian Schnaubelt, MD *(SS)*^2^*****; Lorenz Koller, MD, PhD *(LK)*^1^; Günther Laufer, MD *(GL)*^3^; Arnold Pilz, BA, BA, MD *(AP)*^1^; Niema Kazem, MD *(NK)*^1^; Max-Paul Winter MD *(MPW)*^1^; Barbara Steinlechner, MD *(BS)*^4^; Martin Andreas, MD, PhD, MBA *(MA)*^3^; Tatjana Fleck, MD *(TF)*^3^; Klaus Distelmaier, MD, PhD *(KD)*^1^, Georg Goliasch, MD, PhD *(GG)*^1^; Aurel Toma, MD *(AT)*^1^; Christian Hengstenberg, MD *(CH)*^1^; Alexander Niessner, MD, MSc *(AN)*^1^

*** both authors contributed equally to this work**

**Affiliations:**

^1^ Division of Cardiology, Department of Internal Medicine II, Medical University of Vienna, Austria

^2^ Department of Emergency Medicine, Medical University of Vienna, Austria

^3^ Division of Cardiac Surgery, Department of Surgery, Medical University of Vienna, Austria

^4^ Department of Anesthesia, General Intensive Care and Pain Management, Medical University of Vienna, Austria

**Supplementary Table: Baseline Characteristics for the entire study population and stratified in days of pre-operative hospitalization**

**Total Study Population ≤ 2 days 2 – 7 days > 7 days p-value**

Pre-Operative Stay, days (IQR) 6 (2-12) 1 (1-2) 5 (4-6) 14 (10-16) **<0.001**

Post-Operative ICU Stay, days (IQR) 2 (1-5) 1 (2-4) 2 (1-4) 2 (3-6) **0.004**

Post-Operative Normal Ward Stay, days (IQR) 7 (6-10) 7 (5-8) 7 (6-9) 9 (7-16) **<0.001**

Total Stay, days (IQR) 16 (11-26) 10 (8-12) 14 (12-17) 28 (22-37) **<0.001**

**Patient Characteristics**

Age, years (IQR) 69 (61-75) 68 (59-73) 70 (59-75) 72 (64-77) 0.065

Male gender, n (%) 125 (69.2) 37 (66.1) 46 (73.0) 52 (68.4) 0.827

Type of Surgery 0.075

CABG, (%) 64 (32.8) 13 (6.7) 19 (30.2) 32 (42.1)

Cardiac Valve, (%) 86 (44.1) 32 (57.1) 29 (46.0) 25 (32.9)

CABG and Cardiac Valve, (%) 45 (23.1) 11 (19.6) 15 (23.8) 19 (25.0)

Antibiotic Prophylaxis, n (%) 195 (100.0) 56 (100.0) 63 (100.0) 76 (100.0) -

EuroScore II, % (IQR) 2.4 (1.4-6.5) 1.8 (1.1-3.9) 2.1 (1.3-4.0) 3.1 (1.8-5.7) **<0.001**

*EuroScore class* **0.004**

<4%, n (%) 56 (28.7) 42 (75.0) 47 (74.6) 40 (52.6)

4-9%, n (%) 63 (32.3) 9 (16.1) 10 (15.9) 13 (17.1)

>9%, n (%) 76 (39.0) 5 (8.9) 6 (9.5) 23 (30.3)

**Medical History**

Previous Cardiac Surgery, n (%) 14 (7.2) 3 (5.4) 4 (6.2) 7 (9.2) 0.385

Hypertension, n (%) 153 (78.5) 41 (73.2) 50 (79.4) 62 (81.6) 0.259

Diabetes Mellitus Type II, n (%) 59 (30.4) 15 (26.8) 14 (22.6) 30 (39.5) 0.078

Previous Myocardial Infarction, n (%) 55 (28.2) 10 (17.9) 17 (27.0) 28 (36.8) **0.016**

Chronic Kidney Disease, n (%) 40 (20.5) 11 (19.6) 11 (17.5) 18 (23.7) 0.530

Chronic Obstructive Pulmonary Disease, n (%) 45 (23.1) 11 (19.6) 14 (22.2) 20 (26.3) 0.363

Stroke, n (%) 17 (8.8) 5 (8.9) 5 (7.9) 7 (9.3) 0.917

Cancer, n (%) 31 (15.9) 10 (17.9) 11 (17.5) 10 (13.2) 0.447

Frailty, n (%) 25 (12.8) 4 (7.1) 5 (7.9) 16 (21.1) **0.014**

**Routine Laboratory Measures**

C-reactive Protein at admission, mg/dl (IQR) 0.2 (0.1-0.5) 0.2 (0.1-0.6) 0.2 (0.1-0.5) 0.2 (0.1-0.6) 0.727

Maximum C-reactive Protein after Surgery, mg/dl (IQR) 14.3 (8.9-21.2) 13.1 (9.2-20.7) 14.2 (8.8-20.6) 15.9 (8.7-23.4) 0.596

Maximum total Leucocytes after Surgery, (IQR) 13.4 (10.0-18.1) 14.2 (10.2-18.3) 13.7 (11.1-17.4) 13.4 (9.6-18.3) 0.781

**Categorical data are presented as counts and percentages, continuous data as medians and IQRs. Categorical data are analyzed using a test for linear association (Maentel–Haenszel chi-square test), continuous data using Kruskal–Wallis test for testing within the subgroups.**
